# Plant-Based Biostimulants Influence the Agronomical, Physiological, and Qualitative Responses of Baby Rocket Leaves under Diverse Nitrogen Conditions

**DOI:** 10.3390/plants8110522

**Published:** 2019-11-19

**Authors:** Ida Di Mola, Lucia Ottaiano, Eugenio Cozzolino, Mauro Senatore, Maria Giordano, Christophe El-Nakhel, Adriana Sacco, Youssef Rouphael, Giuseppe Colla, Mauro Mori

**Affiliations:** 1 Department of Agricultural Sciences, University of Naples Federico II, 80055 Portici, Italy; 2 Council for Agricultural Research and Economics (CREA)—Research Center for Cereal and Industrial Crops, 81100 Caserta, Italy; 3 Council National Research (CNR), 80055 Portici, Italy; 4 Department of Agriculture and Forest Sciences, University of Tuscia, 01100 Viterbo, Italy

**Keywords:** ascorbic acid, *Diplotaxis erucoides* L., greenhouse conditions, nitrate, nutritional quality, protein hydrolysate, SPAD index, tropical plant extract

## Abstract

Nitrogen is the primary technical means responsible for food production increase, but on the other hand, wise management is needed because its excessive use can have a negative impact on the environment and on green leafy vegetable quality, such as that rocket. Rocket has the characteristics of accumulating nitrate in leaves with possible impacts on human health. In order to overcome this issue, researchers are focusing their attention on the use of alternative means, such as plant biostimulant application. The scope of this study was to assess the effect of legume-derived protein hydrolysate(LDPH) and tropical plant extract(TPE), combined with various doses of nitrogen (0 kg ha^−1^ non-fertilized; N0); 60 kg ha^−1^ (sub-optimal; N1); 80 kg ha^−1^ (optimal; N2); and 100 kg ha^−1^ (supra-optimal; N3)), in order to reduce nitrogen use, boost yield, and enhance the chemical and nutritional value of leaves without significantly accumulating nitrate. Both vegetal-based plant biostimulants enhanced plant growth, boosted the marketable yield (especially at N0 and N1 levels, by 38.2% and 28.2%, respectively, compared to the non-treated control), and increased the SPAD (Soil Plant Analysis Development) index and leaf pigments content, such as chlorophyll and carotenoids, especially in treated-LDPH rocket. The plant-based biostimulants also produced a major amplification in lipophilic antioxidant activity (+ 48%) and total ascorbic acid content (average + 95.6%), especially at low nitrogen fertilization levels, and maintained nitrate content under the legal European Comission limits.

## 1. Introduction

In the last decades, world food demand has been increasing due to global population growth. In order to satisfy this concern, growers around the globe are intensifying food crop production by increasing the use of resources, in particular, fertilizers [1]. A large number of crops, including leafy vegetables, require large quantities of nitrogen to reach maximal productivity, notwithstanding that high nitrogen availability is not correlated with higher quality of produce. Conversely, excessive nitrogen fertilization can trigger superfluous vegetative growth, making plants prone to pathogen attacks [1]. Furthermore, uncurbed nitrogen presence usually induces nitrate accumulation in leafy vegetables to levels that exceeds the EU regulation limits [2,3]. Exorbitant nitrogen fertilizer rates can also have deleterious impacts on human health [4,5,6] and on the environment through nitrate leaching into water resources and greenhouse gas emissions, such as nitrous oxide [7]. In fact, after intake into the human body, nitrate can be converted into nitrite, and the latter can cause methemoglobinemia or be used to produce the cancer-causing compounds nitrosamine and nitrosamide [8,9].

Among leafy vegetables, rocket production for use in ready-to-eat salads is increasing in response to market demand. Rocket belongs to the Brassicaceae family and it is known for diverse plant metabolites such as polyphenols, ascorbic acid, and glucosinolates [10,11,12]; however, as is the case in other leafy vegetables, rocket is known as a nitrate hyper-accumulating species. Nevertheless, nitrate accumulation depends on nitrogen availability, but also on specific environmental conditions, such as low radiation, which reduces the nitrate reductase activity [13,14,15]. In order to overcome this issue, the scientific community is focusing its attention especially on the accurate management of nitrogen fertilization, also through the use of alternative methods, such as natural biostimulants, for stimulating early growth and yields.

Plant biostimulants are extracted from organic fresh substances accommodating bioactive compounds. Biostimulants mainly include minerals, humic compounds, vitamins, chitin/chitosan, amino acids, and poly- and oligosaccharides [16,17,18].

The mechanisms activated by biostimulants are still not completely known, and therefore they are under intensive investigations [19,20,21]. Biostimulants act either directly on plant metabolism and physiology or indirectly by enhancing soil-conditions [22] by affecting its microflora that can positively influence plant growth. Nonetheless these natural products modify root conformations and boost their development [16,23,24,25]. Based on the biostimulant composition and purpose of use, the application can be foliar or by irrigation [26]. Furthermore, plant biostimulants act differently on different species and/or cultivars of the same species, and their action is environmentally-, dosage-, and application time-dependent [26]. Such variability of effects can impede generalization.

Biostimulant use has been on the rise and their implementation is gaining popularity in sustainable agriculture. Utilization of these natural products triggers numerous physiological processes that stimulate plant growth and tolerance to abiotic stresses, but simultaneously reduce fertilizer application by improving plant nutrient use efficiency without collateral effects on yield and quality [26].

Our hypothesis is that plant-based biostimulant application can improve produce quality by enhancing the antioxidant content and leaf pigments and trigger plant performance by stimulating root growth. However, the abundance of available products in the market, such as fertilizers, growth regulators, and biostimulants, can negatively influence growers’ reasonable choices and applications, leading to disappointing outcomes [27,28]. In view of this approach, the purpose of the present study was to verify the potential of two vegetal-based biostimulants, combined with various doses of nitrogen, on plant growth parameters, yield, biochemical parameters, and chemical and nutritional value of baby rocket leaf grown under plastic tunnel conditions.

## 2. Results

### 2.1. Influence of N Fertilization Levels and Plant-Based Biostimulants on Yield and Plant Growth Parameters

The findings concerning total and single harvest marketable fresh yields of baby rocket leaf are reported in Table 1. A significant interaction between N levels (N) and biostimulant application (B) was observed for the three harvest dates as well as for total fresh yield. Irrespective of biostimulant application, the marketable fresh yield of rocket was positively affected by increasing N fertilization levels, reaching the higher value at N3 (100 kg N·ha^−1^) in all three harvests (Table 1) but progressively decreasing from the first to the third harvest.

In particular, at the first harvest, marketable yields of N2 and N3 plants (optimal and supra-optimal levels of nitrogen) were similar, and no significant differences between treated and control plants were recorded (Table 1). Contrarily, in the second and third harvest dates, at optimal and supra-optimal levels of nitrogen, the plants sprayed with the two biostimulants exhibited higher fresh yield values than control plants, with no significant difference between legume-derived protein hydrolysates (LDPH) and tropical plant extract (TPE) (Table 1).

No significant interaction between N fertilization rates and biostimulant application was recorded for plant growth parameters: leaf area index (LAI), leaf succulence, and specific leaf weight (SLW) (Table 2). Particularly, the plant growth parameters were highly influenced by nitrogen fertilization levels and to a lesser extent by the biostimulant application (Table 2).

When averaged over biostimulant application, the leaf area index and leaf succulence showed a similar trend to marketable fresh yield with increasing nitrogen levels from 0 to 100 kg ha^−1^. Moreover, from the first to the third harvest dates, the differences between the N treatments were more pronounced. The highest values of SLW were recorded in untreated rocket plants.

Irrespective of N fertilization, the foliar application of plant-based biostimulants (i.e., LDPH and TPE) enhanced the leaf surface expansion by 66% and 80%, in the second and third harvest dates, respectively, in comparison to control, with no significant differences between the two commercial products. Additionally, for leaf succulence and SLW, no differences between the two biostimulants were recorded; the increase in succulence over control treatment was 5.6% and 4.1% in the second and last harvest, respectively (Table 2).

### 2.2. Influence of N Fertilization Levels and Plant-Based Biostimulants on SPAD index and Leaf Colorimetry

Soil Plant Analysis Development (SPAD) index was significantly affected by both factors (nitrogen-N and biostimulant application-B), but without significant interaction N × B (Table 3). When averaged over biostimulant application, the increasing nitrogen levels positively affected the SPAD index, with the highest values recorded in the N3 fertilization level, irrespective of the harvest date (Table 3). Nitrogen doses determined an increase of 16.0%, 27.0%, and 33.9% (average of the three harvests) in N1, N2, and N3 plants, respectively, over non-fertilized plants. Moreover, irrespective of nitrogen fertilization, the foliar application of biostimulants also highlighted an increase of SPAD index (+ 9.3% mean value of the two biostimulants in the three harvest dates) in comparison to untreated plants, but with differences between the two commercial biostimulants only at the third harvest, where the LDPH exhibited the best performance.

Among the physical properties that may orient consumer choices is the produce aspect, particularly the color [29]. In our study, the leaf colorimetric *Commission internationale de l’éclairage* (CIELAB) parameters were only affected by fertilization levels, with no significant effect of biostimulant application nor N × B interaction (Table 4). Notably, augmenting N fertilization levels from 0 to 100 kg ha^−1^ decreased a* values, therefore increasing the green intensity, and increased b* values (Table 4). Both leaf colorimetric traits (a* and b*) decreased from the first to the last harvest. For both parameters, the differences between the three fertilization levels were stronger in the second and especially in the third harvest.

### 2.3. Influence of N Fertilization Levels and Plant-Based Biostimulants on Nitrate Accumulation and Biochemical Parameters

Nitrate content, total chlorophyll, chlorophyll a and b, and carotenoids were affected by both nitrogen levels (N) and biostimulant application (B), but not by their interaction (Table 5). Nitrate content is essential for leafy vegetable quality. In the present paper, nitrate accumulation in leaves increased linearly when N fertilization levels increased from 0 to 100 kg ha^−1^, with the highest value set in N3 fertilized plants.

Additionally, chlorophyllous pigments, particularly total chlorophyll, chlorophyll a and b, and carotenoid content were positively influenced by nitrogen level increases, especially under N2 and N3 regimens (Table 5). Furthermore, when averaged over N fertilization, the foliar application of biostimulants elicited an increase in chlorophyll and carotenoid content with the highest value recorded in plants treated with the LDPH product (Table 5).

### 2.4. Influence of N Fertilization Levels and Plant-Based Biostimulants on Lipophilic Antioxidant Activity and Total Ascorbic Acid

**Figure 1:**
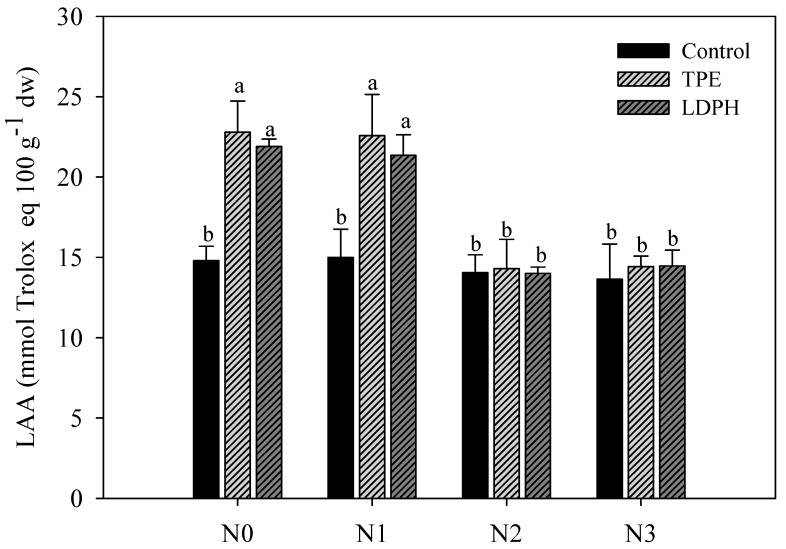
Lipophilic antioxidant activity (LAA) in relation to N fertilization rates (N0 = 0, N1 = 60, N2 = 80, and N3 = 100 kg ha^−1^) and biostimulant application (Control, tropical plant extract (TPE): Auxym and legume-derived protein hydrolysate (LDPH): Trainer). Different letters indicate significant differences at *p* ≤ 0.05.

**Figure 2:**
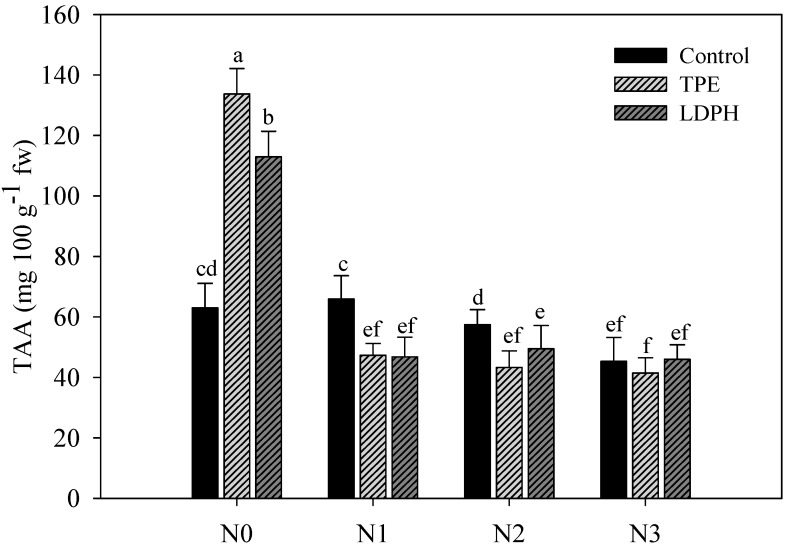
Total ascorbic acid (TAA) in relation to N fertilization rates (N0 = 0, N1 = 60, N2 = 80, and N3 = 100 kg ha^−1^) and biostimulant application (Control, tropical plant extract (TPE): Auxym and legume-derived protein hydrolysate (LDPH): Trainer). Different letters indicate significant differences at *p* ≤ 0.05.

The highest values of lipophilic antioxidant activity (LAA) were recorded in biostimulant-treated rocket at both N0 and N1 treatments, with no significant difference between the two commercial biostimulants (Figure 1). On the other hand, the marked effect of vegetal-based biostimulants on total ascorbic acid (TAA) was recorded in non-fertilized treatments. Particularly, foliar application of TPE and LDPH elicited a significant increase (112.0% and 79.2%, respectively) of TAA content compared to untreated rocket plants (Figure 2).

## 3. Discussion

The use of natural plant biostimulants including LDPH and TPE have been proven, when applied at low concentrations, to boost plant growth, yield, and quality, and to increase tolerance to abiotic stressors [30,31]. In the current study, the stimulation effect of the two plant-based biostimulants was more evident in the non-fertilized (N0) and sub-optimal (N1) treatments. Particularly, the biostimulant application led to significant yield increases in rocket sprayed with LDPH and TPE in comparison to untreated plants, (by 38.2% and 28.2% for N0 and N1 treatments, respectively) with no significant differences between the two commercial products. Positive effects of biostimulant application (TPE and LDPH) on fresh yield of rocket were also reported by Caruso et al. [30] and by several authors on a wide range of fruit and leafy vegetables grown under greenhouse conditions, such as baby leaf lettuce [31], tomato [32], zucchini squash [33], and spinach [34]. Similar to the effects on rocket yield, the increase in LAI recorded in the present work due to biostimulant application was more pronounced than those reported by Rouphael et al. [34] and Caruso et al. [30] on spinach and perennial wall rocket, showing a species-specific response [35], especially when the same two commercial plant biostimulants were tested. Therefore, our findings highlight the need to investigate further this issue for elucidating the physiological and molecular mechanism(s) behind these stimulation effects. In addition, several authors have pointed out that biostimulant application can activate a transduction signal pathway through elicitation of endogenous phytohormone synthesis (i.e., auxin- and gibberellin-like activities), thus leading to a higher yield [36,37]. Moreover, the stimulation of LAI and succulence could be due to the increase of root biomass, volume, length, and higher branching triggered by biostimulant application, which enhanced nutrient uptake/translocation/assimilation, helping in a major nitrogen uptake efficiency and utilization, and consequently higher agronomical performance [33,34,38,39].

The leaf SPAD measurements recorded in LDPH- and TPE-treated plants were significantly higher than in untreated rocket plants. Our data obtained on the SPAD index are consistent with those mentioned for many leafy vegetable species, such as lettuce, perennial wall rocket, jute, and spinach [30,34,40,41]. Two different mechanisms could have caused the higher SPAD values: (i) enhanced N uptake efficiency, and (ii) limited chlorophyll degradation and leaf senescence [36,42,43]. Indeed, this index is considered a major indicator of green pigment biosynthesis efficiency, and thus improved crop outcomes.

Nitrate content is essential for the quality of fresh leafy vegetables, in particular rocket, which is considered a hyper-accumulator species. In the current study, nitrate content in all treatments was below the European Union upper limits for safe rocket marketing EU No 1258/2011 of 6000 to 7000 mg NO_3_^−^ kg^−1^ for wild and salad rocket (depending on growing season and cultivation conditions). Moreover, irrespective of nitrogen fertilization levels, the foliar application of biostimulants determined a significant increase of nitrate content (avg. 94.0%) in rocket leaves sprayed with LDPH and TPE, without violation of EU nitrate limits. The higher accumulation of nitrate in biostimulant-treated plants has been observed previously by several authors [44,45], probably due to a more developed root system (in terms of biomass and root branching), which may have increased nitrate uptake and translocation to the shoots.

The different changes induced by biostimulant application on agronomical traits (yield and LAI) may be reflected at the biochemical level. This was the case in the current experiment, since the beneficial response of plant biostimulant application, in particular LDPH, on chlorophyllous pigment has been recorded also in baby lettuce, eggplant, and corn [40,46,47,48], probably due to amino acid abundance in the LDPH-treated plants, which help to enhance chlorophyll pigment content and augment net photosynthesis rate.

Leafy vegetables are considered an important sources of antioxidant molecules. In the current study, lipophilic antioxidant activity (LAA) and total ascorbic acid (TAA) were significantly affected by N × B interaction. The importance of antioxidant activity, as a functional quality parameter of food, is related to the beneficial effects of antioxidant molecules (hydrophilic and lipophilic) on human health, due to their role in delaying or/inhibiting oxidative damage, hence evading a broad range of diseases [49,50,51,52,53]. Irrespective of biostimulant application, the rocket plants in the optimal (N2) and supra-optimal (N3) treatments were characterized by the lowest quality traits (i.e., lowest LAA). Similar to our findings, Wang and co-workers [54] reported that a high level of nitrogen can result in damage in terms of commercial, nutritional, and functional quality of fruit and leafy vegetables. Furthermore, it is notable that stress conditions, such as low nitrogen input, may stimulate the antioxidant system leading to an increase in antioxidant molecule production in plants [39].

Concerning the effects of plant biostimulants on leaf quality traits, the plant-based biostimulants also determined a major amplification in LAA (+ 48%) and TAA content (+ 95.6%), especially at low nitrogen fertilization levels. These findings are in line with our previous research on baby leaf lettuce sprayed with three different biostimulants at four different levels of nitrogen, in which we found an LAA increase in non-fertilized and low-fertilized regimens [30]. The positive effects of plant biostimulant application on synthesis of antioxidant molecules can be attributed to two putative mechanisms: (1) activation of key enzymes activation that are involved in cell antioxidant homeostasis, and (2) an increase of macro and micronutrients assimilation in plants treated with biostimulants that can play a part in amino acid synthesis such as phenylalanine and tyrosine [55,56].

## 4. Materials and Methods

### 4.1. Growing Conditions, Rocket Cultivar, and Experimental Design

During the 2018 spring season, an experiment was conducted at the experimental site of “Gussone Park” of the University of Naples-Federico II (40°48.870’ N; 14°20.821’ E; 70 m a.s.l.; in Portici, Naples, Southern Italy).

The plastic tunnel was open on both sides and covered on top by polyethylene. The rocket plants were cultivated in large lysimeters (0.70 m diameter and 0.60 m depth). The soil was sandy (69.1% coarse sand, 21.9% fine sand, 4.5% silt, and 4.5% clay) with a pH of 6.6, an electrical conductivity of 0.68 dS m^−1^, an organic matter of 2.6% (w:w), a total N of 1.1 g kg^−1^ (Kjeldhal method), 127.2 mg kg^−1^ of P (Olsen method), and 471.8 mg kg^−1^ of K (Tetraphenylborate method).

The baby rocket leaf (*Diplotaxis erucoides L.*) cultivar ‘Reset’ (Maraldi Sementi S.r.l., Cesena, Italy) was the tested crop. This cultivar is widely used by leafy vegetable growers due to its good adaptability to different environmental conditions.

The experimental design was a factorial combination of four fertilization levels of nitrogen (N) and three treatments of biostimulants (B) (the untreated control did not receive any product). The N levels were: 0 kg ha^−1^ (non-fertilized control—N0); 60 kg ha^−1^ (N1—sub-optimal level); 80 kg ha^−1^ (N2—optimal level); and 100 kg ha^−1^ (N3—supra-optimal level). The biostimulants used were an untreated control, tropical plant extract (TPE), and legume-derived protein hydrolysate (LDPH). Treatments were arranged in a randomized complete-block design and replicated three times each.

### 4.2. Nitrogen Fertilization Management and Biostimulant Characteristics and Application

A density of 3500 plants per square meter (about 0.8 g m^−2^ of seeds) was adopted to hand seed baby rocket leaf on 16 April 2018. Calcium nitrate (26% N) was the nitrogen source; it was distributed as follows: (i) first treatment 50% of the total amount applied ten days after sowing; (ii) second treatment 25% after the first harvest; (iii) third treatment 25% after the second harvest. During the growing cycle, lysimeters were irrigated with an amount of water corresponding to the complete replacement of water lost by evapotranspiration.

The two commercial biostimulants, legume-derived protein hydrolysate-LDPH (Trainer^®^) and tropical plant extract-TPE (Auxym^®^) were supplied by Italpollina S.p.a. (Rivoli Veronese, Italy). An enzymatic hydrolysis process was used to produce the LDPH that contained free amino acids and peptides, carbohydrates, and mineral nutrients with the following percentages of 75%, 22%, and 3%, respectively [57,58]. A fermentation process of tropical plants such as hibiscus was used to produce the TPE biostimulant, which contained free amino acids/peptides, carbohydrates, mineral nutrients, vitamins, and phytohormones with the following percentages of 54%, 17%, 23%, 6%, and 0.22%, respectively, as reported in detail by Rouphael et al. [59] and Caruso et al. [60].

The treatments with biostimulants started 21 days after the sowing; a total of four applications were applied on a weekly basis. A solution containing 3 mL L^−1^ of LDPH and 2 mL L^−1^ of TPE-based biostimulant was used to spray rocket leaf plants, and control plants were sprayed with water.

### 4.3. Morphological Parameters and Leaf Colorimetry

Rocket plants were harvested at three different dates during the growing period (on 14 May, 30 May, and 13 June). A sample area of 0.40 × 0.40 m was harvested from each lysimeter to avoid the edge effect. At all harvest dates, leaf area of rocket was measured with an electronic leaf area meter (Li-Cor3000, Li-Cor, Lincoln, NE, USA) and then the leaf area index (LAI) was calculated based on the crop density. The marketable yield was determined and expressed in tons per hectare; a sample was oven dried at 70 °C until reaching a steady weight; then samples of dried leaves were used for determining leaf succulence (mg water cm^−2^), specific leaf weight (mg dw cm^−2^), and nitrate content determination.

The color space parameters, L*, a*, and b*, were determined by a Minolta CR-300 Chroma Meter (Minolta Camera Co. Ltd., Osaka, Japan) by measuring on the upper side of ten leaves per replicate. On ten undamaged and fully developed leaves, the Soil Plant Development Analysis (SPAD) index was also measured using a portable chlorophyll meter (SPAD-502, Konica Minolta, Tokyo, Japan). After harvest, leaf samples from all treatments were frozen in liquid nitrogen and then lyophilized with the means of a Crist, Alpha 1-4 (Osterode, Germany), to be stored at −80 °C for future chemical analysis.

### 4.4. Lipophilic Antioxidant Activity and Total Ascorbic Acid Analysis

The lipophylic antioxidant activity was determined on 200 mg of freeze-dried material using methanol. It was assessed spectrophotometrically (Hach DR 2000, Hach Co., Loveland, CO, USA) according to the methods of Re et al. [61], and the absorbance of the solution was measured at 734 nm. Lipophylic antioxidant activity was expressed as mmol of Trolox per 100 g of dry weight (dw) [62].

The total ascorbic acid (TAA) was also assessed spectrophotometrically according to the protocol stated by Kampfenkel et al. [63]. The solution absorbance was measured at 525 nm.

### 4.5. Chlorophyll, Carotenoid, and Nitrate Analysis

Rocket leaf chlorophyll and carotenoid content was also measured by means of a spectrophotometer after the extraction with ammoniacal acetone according to the method described by Wellburn [64], whereas the nitrate content was assessed based on the protocol of Sah [65]. Solution absorbances were measured at 662 and 647 for chlorophyll a and b, at 470 nm for carotenoids, and at 550 nm for nitrate. Total chlorophyll, chlorophyll a and b, and carotenoids were expressed as mg g^−1^ fw, whereas nitrate content was expressed in mg kg^−1^ fw.

### 4.6. Statistical Processing

Morphometric, physiological, and qualitative data were subjected to variance analysis (two-way ANOVA) using SPSS 21 for Windows 2007. In case of interaction between the two tested factors, mean comparisons were made between the N rate × biostimulant application, and letters separated by Duncan’s test (significance level 0.05) were used to denote comparisons between these one-way means. On the other hand, when non-significant interaction was recorded between the two tested factors, the letters separated by Duncan’s test (significance level 0.05) were used to denote statistical differences between the main effects.

## 5. Conclusions

Fertilizers, in particular nitrogen, are a crucial part of agriculture, being a tool farmers use to enhance yield and guarantee continuous productivity all year long [66,67]. Simultaneously, their synthetic/chemical characteristics can influence human health and ecosystems. Therefore, finding sustainable means of production, such as use of plant biostimulants to boost yield and quality under both optimal and sub-optimal conditions, is an urgent need for horticulturists and scientists. In this study, the foliar application of vegetal-based biostimulants stimulated plant growth and boosted the marketable yield. The better agronomical performance was attributed to an improved physiological (higher SPAD index) and biochemical (higher chlorophyll and carotenoids content) status of plants, especially with LDPH use. Interestingly, foliar application of biostimulants enhanced the functional quality of baby rocket leaf in terms of lipophilic antioxidant activity and total ascorbic acid content, especially under a sub-optimal nitrogen regimen. The results displayed in the current study will contribute to understanding the biostimulant effects behind the variation in yield and quality of rocket and will assist the leafy vegetable industry in identifying optimum biostimulant–nitrogen fertilization regimens for achieving higher productivity and functional quality.

## Figures and Tables

**Table 1:** Baby rocket leaf marketable yield at different harvest dates in relation to N fertilization rates (N0 = 0, N1 = 60, N2 = 80, and N3 = 100 kg ha^−1^) and biostimulant application (Control, tropical plant extract (TPE): Auxym and legume-derived protein hydrolysate (LDPH): Trainer).

Treatments		I Harvest	II Harvest	III Harvest	Total
		(t ha^−1^)			
N0	Control	6.35 e	1.73 g	2.01 e	10.09 f
	TPE	7.75 d	3.56 ef	2.40 de	13.71 e
	LDPH	8.10 d	3.15 f	2.93 cd	14.18 de
N1	Control	10.15 c	3.64 ef	2.10 e	15.89 d
	TPE	10.73 bc	4.35 ce	5.09 b	20.17 c
	LDPH	10.93 b	4.53 cd	5.11 b	20.57 c
N2	Control	11.65 a	3.92 df	3.19 c	18.76 c
	TPE	11.80 a	7.38 b	5.49 b	24.67 b
	LDPH	11.87 a	7.15 b	5.08 b	24.10 b
N3	Control	11.73 a	4.82 c	3.57 c	20.13 c
	TPE	11.57 a	9.04 a	7.18 a	27.79 a
	LDPH	11.70 a	8.35 a	7.40 a	27.44 a
**Significance**					
Nitrogen (N)		**	**	**	**
Biostimulants (B)		NS	*	*	*
N × B		**	**	**	**

NS, *, ** Non-significant or significant at *p* < 0.05 and 0.01, respectively. Different letters within each column indicate significant differences at *p* ≤ 0.05.

**Table 2:** Baby rocket leaf plant growth parameters (leaf area index (LAI), leaf succulence and specific leaf weight (SLW)) at different harvest dates in relation to N fertilization rates (N0 = 0, N1 = 60, N2 = 80, and N3 = 100 kg ha^−1^) and biostimulant application (Control, tropical plant extract (TPE): Auxym and legume-derived protein hydrolysate (LDPH): Trainer).

Treatments	LAI			Succulence			SLW		
	I Harvest	II Harvest	III Harvest	I Harvest	II Harvest	III Harvest	I Harvest	II Harvest	III Harvest
				*(mg H_2_O cm^−2^)*			*(mg d.m. cm^−2^)*		
**Nitrogen (N)**									
N0	3.5 c	1.0 d	0.9 c	51.5 b	66.9 b	65.2 c	4.4 a	6.0 a	7.4 a
N1	4.6 b	1.5 c	1.5 b	55.9 a	68.8 b	68.7 bc	4.4 a	5.6 b	5.4 b
N2	5.2 a	2.0 b	1.6 b	55.8 a	74.2 a	70.6 ab	4.1 b	5.6 b	5.5 b
N3	5.1 a	2.4 a	2.0 a	56.5 a	74.7 a	73.1 a	4.0 b	5.6 b	5.8 b
**Biostimulants (B)**									
Control	4.5	1.2 b	1.0 b	54.5	68.6 b	67.9 b	4.2	5.8	6.8 a
TPE	4.7	2.1 a	1.8 a	55.1	71.9 a	70.8 a	4.3	5.7	5.7 b
LDPH	4.7	1.9 a	1.8 a	55.6	73.0 a	70.6 a	4.1	5.6	5.7 b
**Significance**									
N	*	*	*	*	**	**	*	**	**
B	NS	*	*	NS	*	*	NS	NS	**
N × B	NS	NS	NS	NS	NS	NS	NS	NS	NS

NS, *, ** Non-significant or significant at *p* < 0.05 and 0.01, respectively. Non-significant interaction was recorded between the two tested factors; the letters separated by Duncan’s test (significance level 0.05) were used to denote statistical differences between the main effects.

**Table 3:** Baby rocket leaf plants Soil Plant Analysis Development (SPAD) index at different harvest dates in relation to N fertilization rates (N0 = 0, N1 = 60, N2 = 80, and N3 = 100 kg ha^−1^) and biostimulant application (Control, tropical plant extract (TPE): Auxym and legume-derived protein hydrolysate (LDPH): Trainer).

Treatments	SPAD Index		
	I Harvest	II Harvest	III Harvest
	*(%)*		
**Nitrogen (N)**			
N0	22.9 d	31.8 d	23.8 d
N1	27.4 c	34.1 c	29.3 c
N2	29.1 b	36.4 b	33.2 b
N3	31.1 a	37.5 a	35.2 a
**Biostimulants (B)**			
Control	26.1 b	32.6 b	28.7 c
TPE	28.1 a	35.8 a	30.7 b
LDPH	28.6 a	36.4 a	31.7 a
**Significance**			
N	**	**	**
B	*	*	**
N × B	NS	NS	NS

NS, *, ** Non-significant or significant at *p* < 0.05 and 0.01, respectively. Non-significant interaction was recorded between the two tested factors; the letters separated by Duncan’s test (significance level 0.05) were used to denote statistical differences between the main effects.

**Table 4:** Baby rocket leaf plants hunter color parameters (L*, a*, and b*) at different harvest dates in relation to N fertilization rates (N0 = 0, N1 = 60, N2 = 80, and N3 = 100 kg ha^−1^) and biostimulant application (Control, tropical plant extract (TPE): Auxym and legume-derived protein hydrolysate (LDPH): Trainer).

Treatments	I Harvest			II Harvest			III Harvest		
	L*	a*	b*	L*	a*	b*	L*	a*	b*
**Nitrogen (N)**									
N0	42.83	–16.20 b	25.15 b	39.82	−14.26 c	20.38 b	41.73	–13.01 c	20.39 c
N1	42.96	–16.85 a	25.69 a	39.74	–14.75 b	20.68 b	41.81	–13.30 bc	20.71 bc
N2	42.96	–16.90 a	25.96 a	39.77	–14.91 ab	21.23 a	41.63	–13.69 ab	20.91 ab
N3	42.96	–17.07 a	27.15 a	39.83	−15.07 a	21.40 a	41.66	–13.85 a	21.30 a
**Biostimulants (B)**									
Control	42.87	–16.75	26.20	39.71	–14.64	20.84	41.74	–13.46	20.83
TPE	43.00	–16.72	26.33	39.79	–14.77	20.94	41.67	–13.56	20.89
LDPH	42.91	–16.79	26.18	39.87	–14.83	20.98	41.72	–13.38	20.77
**Significance**									
N	NS	*	**	NS	**	**	NS	**	**
B	NS	NS	NS	NS	NS	NS	NS	NS	NS
N × B	NS	NS	NS	NS	NS	NS	NS	NS	NS

NS, *, ** Non-significant or significant at *p* < 0.05 and 0.01, respectively. Non-significant interaction was recorded between the two tested factors; the letters separated by Duncan’s test (significance level 0.05) were used to denote statistical differences between the main effects.

**Table 5:** Baby rocket leaf plants nitrate, chlorophyll, and carotenoid content as mean value of the three harvests, in relation to N fertilization rates (N0 = 0, N1 = 60, N2 = 80, and N3 = 100 kg ha^−1^) and biostimulant application (Control, tropical plant extract (TPE): Auxym and legume-derived protein hydrolysate (LDPH): Trainer).

Treatments	Nitrate	Chlorophyll a	Chlorophyll b	Total Chlorophyll	Carotenoids
	(mg kg^−1^ fw)	(mg g^−1^ fw)	(mg g^−1^ fw)	(mg g^−1^ fw)	(µg g^−1^ fw)
**Nitrogen (N)**					
N0	1049.3 d	0.741 c	0.464 b	1.205 b	232.0 c
N1	2417.9 c	0.843 bc	0.572 ab	1.415 a	254.0 b
N2	3036.5 b	0.873 ab	0.555 ab	1.427 a	265.0 ab
N3	4417.4 a	0.883 a	0.624 a	1.507 a	280.0 a
**Biostimulants (B)**					
Control	1650.0 b	0.814 b	0.524 b	1.338 b	253.0 b
TPE	3504.2 a	0.784 b	0.507 b	1.291 b	247.0 b
LDPH	2900.1 a	0.907 a	0.630 a	1.537 a	274.0 a
**Significance**					
N	*	*	*	*	**
B	*	*	*	*	*
F × B	NS	NS	NS	NS	NS

NS, *, ** Non-significant or significant at *p* < 0.05 and 0.01, respectively. Non-significant interaction was recorded between the two tested factors; the letters separated by Duncan’s test (significance level 0.05) were used to denote statistical differences between the main effects.
